# Comparison of the effectiveness of the electronic portfolio and online discussion forum methods in teaching professional belonging and ethical behaviors to nursing students: a randomized controlled trial

**DOI:** 10.1186/s12909-022-03677-0

**Published:** 2022-08-15

**Authors:** Reza Baghbani, Mahnaz Rakhshan, Nahid Zarifsanaiey, Reza Nemati, Safieh Daneshi

**Affiliations:** 1 Department of Medical Emergencies, School of Allied Medical Sciences, Bushehr University of Medical Sciences, Bushehr, Iran; 2 Department of Nursing, School of Nursing and Midwifery, Shiraz University of Medical Sciences, Shiraz, Iran; 3 Department of E-Learning in Medical Sciences, Virtual School and Center of Excellence in E-Learning, Shiraz University of Medical Sciences, Shiraz, Iran; 4 Clinical Research Development Center, The Persian Gulf Martyrs Hospital, Bushehr University of Medical Sciences, Bushehr, Iran

**Keywords:** Electronic portfolio, Ethical behaviors, Nursing students, Online discussion forum, Professional belonging

## Abstract

**Background:**

Nursing is a profession that has had many ethical aspects and understanding professional belonging and ethics as a deep and complex process is one of the basic concepts in this field. This study aimed to compare the effectiveness of training professional belonging and ethical behaviors in two methods: electronic portfolio and online discussion forum in nursing students.

**Methods:**

This study is a single-blinded randomized-controlled trial (RCT) with two parallel intervention groups and a third control group. The sample size was 90 selected by block randomization method. The educational contents of professional belonging and ethical behaviors were presented to the participants in two ways: electronic portfolio and online discussion forum. Demographic information form, professional belonging questionnaire, and ethical behaviors questionnaire were applied to collect data. Data were analyzed using SPSS version 24 software. Respectively mean, standard deviation and repeated measured, analysis of variance tests was used in descriptive and analytic statistic. (*P* value < 0.05).

**Results:**

Comparison of the mean score of professional belonging and ethical behavior in the three stages of pretest, immediately after the test and four weeks after the test in all three groups was significant (*P* < 0.001). The control group had a higher mean score of professional belonging immediately after the test (108.18 ± 48.9) compared to the other two groups. Also, the online discussion forum group had a higher mean score on ethical behavior in four weeks after the test (104.2 ± 0.8) compared to the other two groups.

**Conclusion:**

Training based on two methods of the electronic portfolio and online discussion forum increases and enhances the level of ethical behaviors in students. Therefore, the implementation of such methods of training can be useful in improving, promoting, and learning ethical behaviors in nursing students. On the other hand, training based on the two methods had a negative impact on professional belonging. Therefore, it is suggested that future studies be conducted with a greater focus on areas of professional belonging.

**Trial registration:**

This research has been registered in Iranian Clinical Trial Registration Center (IRCT) with registration number "IRCT20180612040063N1" and registration date "16/07/2018".

## Background

Sense of belonging is one of the most important factors of socialization in a person's work environment [1]. According to Maslow, the sense of belonging is one of the basic needs of the individual [2]. Somer considers belonging as the need to be with others and to understand it at different interpersonal levels, having two important consequences; a sense of belonging and dignity. These consequences, in turn, result in gaining a sense of acceptance, a sense of worth, and feeling respected by others [3]. Professional belonging is an important phenomenon and concept for nursing students and people involved in their education. The experience of a sense of belonging is formed in two ways: either actively as a result of individual actions in the environment or passively as a result of group performance. Lack of a sense of belonging can lead to behavioral, emotional, psychological, and physical consequences in the individual [4]. Mattila et al. (2010) in their study concluded that when nursing students experience a sense of acceptance in the group and are seen as equal members, they feel self-fulfilling and satisfied [5]. Nursing students need a sense of professional belonging because it is a necessary concept in the acceptance of the profession by the student, and its positive effects such as a sense of job satisfaction, sense of responsibility, adherence to ethical principles and behaviors, and the like are reflected in the clinical environment [6].

The nursing profession is one of the jobs that has had many distinguished moral aspects from the past time to the present [7]. Understanding ethics is a deep and complex process and one of the basic concepts of nursing [8]. In recent years, despite increasing advances in medical science and new technologies, health-related ethical concerns are on the rise [9, 10]. The emergence of new diseases, the use of modern technology in hospitals, the announcement of brain death, organ transplants, genetic manipulation, and countless other reasons have always led to new scenarios of ethical challenges in providing care [11]. Nurses in clinical settings often face many challenges and situations that require ethical behavior and complex ethical decisions that have a profound effect on their performance; as appropriate ethical behaviors can positively affect the improvement of patients’ conditions [12, 13].

Nursing students, as prospective nurses, must take care of their patients with the right ethical behaviors [14]. Therefore, given the importance of nurses' ethical behavior in decision making, it is necessary to find ways to promote ethical reasoning and nurses' moral growth; Therefore, empowering nurses during their education period is very important [15, 16]. In this regard, the issue of teaching ethics in nursing education has become increasingly important in recent years. Despite the availability and use of many research-based studies on ethics education, there are still controversies among nursing educators about the best methods to teach ethical behaviors [17].

The use of e-learning methods in the educational system is one of the new methods of assessment and learning in which learners achieve educational goals according to their talents [18]. Among these methods used in medical education are two methods of the electronic portfolio and online discussion forum.

Having a high degree of flexibility, the electronic portfolio leads to the assessment of the level of thinking and social skills [19]. In the Oxford Dictionary, Portfolio is defined as “a thin flat case used for carrying documents, drawings, etc.” [20]. But a portfolio is not really a simple tool for gathering documents; rather, it is a useful tool for strengthening general and specific skills, critical thinking and rethinking, connecting theoretical knowledge to practice, and developing knowledge [21]. In this way, individuals make a continuous effort to understand and evaluate what they have learned in order to achieve independence and take responsibility for their learning [22]. The portfolio is increasingly applied as a learning and assessment tool in medical education, and by creating an organized framework, it allows students to interact in activities and have more control over the learning process [23]. Many studies confirm the importance and usefulness of the portfolio method in education [23–25].

Nowadays, higher education needs dynamism and presents its programs in a way that suits the needs of society and the scientific knowledge of each field. The rapid development of information and communication technologies has created new opportunities in the field of planning and implementation of new teaching methods [26]. Web-based education approaches are considered useful for providing quality education, as they have advantages such as quick access and a shift from traditional and teacher-centered education to student-centered approaches [27]. One of the methods of web-based education approaches is the conversation forum. Online environments, such as discussion forums, are commonly applied in higher education for students to use in a variety of group learning activities [28]. According to Williams (2014), the online discussion forum is used by group participation to seek information or solve problems that occur in people's lives [29]. The online discussion forum, as a collective and group learning tool, allows learners to create deep and critical thinking at any time and place, discussing the topic at hand in a free space without fear or shame, and contributing to the learning process of oneself and others [30, 31]. In addition, because communication is symmetrical and based on content and dialogue, participants have more time to answer questions or interact with each other, which provides a high potential for deeper thinking and discussion [32]. Bramer et al. (2020) in a qualitative study entitled “Adult Nursing Students' Experiences of Online Education” found that students preferred access and convenience of using online education to traditional education; however, they considered traditional education more effective than online education in respect to interaction [33]. On the other hand, some studies suggest that the use of online discussion forums has beneficial effects on teaching methods, providing a basis for facilitating the search and exchange of information and a tool for analyzing educational data [34]. Also, the electronic conversations create trust and group cohesion in an educational environment [35].

Few studies have been performed on training professional belonging and ethical behaviors, which have shortcomings. Kruse et al. (2020) conducted a study entitled “Strengthening Student Nurses’ Sense of Belonging through Attendance at a Professional Nursing Conference.” The results showed an increase in students' professional belonging by attending conferences [36]. Zakaria et al. (2016) also found in their study that awareness of nursing ethics and ethical behaviors are strongly correlated with educational programs [37]. Meanwhile, the last two studies have been conducted in the form of face-to-face workshops, which can deprive the participants of the interaction with each other and the opportunity for each person to think about various nursing issues. In a study entitled “Developing Nursing Ethical Competencies Online Versus in the Traditional Classroom”, Trobec et al. (2015) reported that there was no significant difference between online and face-to-face training [38]. In the study, the presence or absence of clinical experiences in the participants was not mentioned. while reports indicate a positive impact of clinical experiences on learning [39].

Due to the nature of the electronic portfolio and online discussion forum methods and the abstract and complex concept of ethics, as well as the shortcomings of ethics training programs for nurses and the lack of empirical studies that document these two educational approaches in ethics education, and based on the studies on stressful challenges and ethical issues and their impact on the quality of nursing caregivers [40, 41], conducting research in this field is considered significant. On the other hand, the software flexibility of both online discussion forum environment and electronic portfolios and the existence of features such as determining user access levels, grouping users separately, stability of comments and information sent by each user, as well as easier access to these virtual environments make these two platforms more effective for training programs of professional belonging and ethical behaviors.

### Aim of the study

This study aimed to compare the effectiveness of the two methods of the electronic portfolio and online discussion forum in teaching professional belonging and ethical behaviors to nursing students.

### Study hypotheses

1. Nursing students trained through online discussion forum have a higher level of professional belonging and ethical behaviors than those trained using face-to-face.
2. Nursing students trained through electronic portfolio have a higher level of professional belonging and ethical behaviors than those trained using face-to-face.

## Methods

### Design

This study is a single-blinded randomized-controlled trial (RCT) with two parallel intervention groups and a third control group.

### Settings

The present study had been conducted in 2018 three months (The total time of the study including intervention and follow-up, from September to December) in the School of Nursing and Midwifery of Shiraz University of Medical Sciences. The university is the largest scientific center for advanced e-learning in medical sciences in southern Iran and has a dedicated virtual faculty in this field [42]. The total number of nursing students was 1091 (undergraduate 1031 and 60 postgraduate), at the time of the study.

### Sampling and randomization

First, the list of students was taken from the faculty. Then, using their contact information, we could invite some of them to participate in this research through WhatsApp software or by phone calls. Some others were invited by visiting them in their classes or internship environments. Then, to homogenize the groups by random allocation, they were divided into 4 categories based on the level of education (bachelor and master) and gender (male and female): master-male, master-female, bachelor-male, and bachelor-female. Then, in a block randomization method and using a table of random numbers, in each category the samples were divided into 3 groups: electronic portfolio, online discussion forum, and control group (without intervention). Participants did not know which students were included in each group.

### Participants

The research population was the nursing students of the School of Nursing and Midwifery of Shiraz University of Medical Sciences. Sampling was done among students who had started internships and practical training in the clinic, according to the concepts of professional belonging and ethical behaviors based on socialization in the work environment, feelings of acceptance in the group, and ethical challenges and issues.

Inclusion criteria were the consent to enter the study, being undergraduate nursing students in semesters 4 to 8 and postgraduate nursing students in semesters 2 to 6, as well as easy access to the Internet. The contents of the virtual training of ethical behavior and professional belonging were taught simultaneously to both undergraduate and postgraduate nursing students. Exclusion criteria were unwillingness to continue participating in the study, being absent from the introductory session on the educational method, inactivity and not participating in related topics. After the objectives of the project were explained to the students, those who were willing to participate in the study and benefited from the inclusion criteria entered the study by completing the informed consent form.

### Sample size calculation

The researchers began their research with a pilot study and calculated the sample size. Accordingly, in order to detect an effect size f = 0.28 with 90% power and alpha = 0.05, number of groups = 3, number of measurements = 3, correlation among repeated measures = 0.156, no sphericity correction ε = 0.557, G*Power suggests we would total sample size *N* = 72 in a repeated measure, within factors test. Considering 20% dropout, the total sample size was 90 with 30 cases in each group.

### Research methods and data quality control

#### Preparing the educational environment

Consulting the e-learning and technology experts in the virtual faculty of Shiraz University of Medical Sciences, and reviewing the available software, Mahara, was selected. Mahara is one of the most popular open-source systems and a user-based environment in which users can easily store, edit, and manage their manuscripts. It also contains blogs and social networking systems such as online conversation forums that can create online communities by connecting users to each other.

#### Preparation of educational materials

The appropriate educational contents in the field of professional belonging and ethical behaviors according to the opinion of the research team experts were provided by searching the valid databases and up-to-date articles. Collecting the materials and compiling the concepts and content of professional belonging and ethical behavior were approved at each stage under the supervision of the research experts. Then, a schedule was designed separately for each experimental group according to the length of the intervention and the opinion of the research team experts and was presented to the students in either educational space. The final approval of the educational content was done by the ethics professors of this research.

All nursing students have only one course related to nursing ethics and professional aspects based on the nursing curriculum in the 2nd semester of the bachelor's degree. The course mostly covers the historical aspects and theoretical foundations of ethics in nursing and its basic principles and perspectives. Master’s students have no additional course in this regard. Also, in the nursing curriculum, there is no specialized course in the field of increasing professional belonging. Therefore, considering university courses, the level of knowledge is the same in bachelor's and master's degrees.

Thus, in the present study, it seemed necessary to provide practical and scenario-oriented contents by experts for both groups of students. Separate contents were prepared for either of the two concepts of ethical behavior and professional belonging. The contents were presented in both groups of online discussion forum and electronic portfolio to all students of the two intervention groups, in the same way and at the same time.

#### Planning and implementation of educational intervention

First, the pre-test questionnaire was distributed to all participating students, and the data were collected. Then, the participants were divided into three groups of 30 participants, based on the randomization method, two of which entered the Mahara space. One group were given access to the electronic portfolio and the other group were given access to the online discussion forum.

It should be noted that both training methods were presented on a website and based on the research team and the research technical engineer, the training site was designed in such a way that the members of the two test groups could not establish any communication with each other in the training environment. Then, for each group, a separate online session for 2 h, instruction on how to use the electronic portfolio and online discussion forum was given, and students' questions and ambiguities were answered and clarified. The duration of the intervention was determined 5 weeks, based on similar studies [43, 44] and research advisors. The participants could choose to work at any time of the day during these 5 weeks in the designated educational space. According to the educational content provided by the professors in the field of principles of nursing ethics, scenarios were designed and the students were encouraged to comment on them and raise questions during the intervention at the beginning of each week. All scenarios proposed for the students were similar.

In the e-portfolio group, at the beginning of each week, a portion of the content assigned to the professional belonging and ethical behaviors was presented to the students by asking questions and scenarios, and the students sent their questions and comments to the professor and received feedback so that learning process happens individually by rethinking.

In the online discussion forum group, in addition to expressing their opinions, the students also criticized the opinions of their peers, and the professor supervised the students' opinions and topics as a guide. In this group, according to the same schedule, at the beginning of each week, questions and scenarios were posted in the forum, and the members expressed their opinions and criticized each other's opinions.

At the end of the course, based on the experiences of expert professors, appropriate summarizing and dealing with each challenge mentioned in the scenarios, were done in each group and in the platform. Then, immediately after the intervention and four weeks after the intervention, through the educational site, the students in both the intervention and control groups were provided with the questionnaires to be completed.

The control group received conventional training based on the nursing curriculum with no special training or intervention. But at the end of the study, the students of the control group received the educational content that was presented to the 2 intervention groups in the form of educational booklets.

It should be noted that during the intervention, the researchers encouraged the students to cooperate actively, through phone calls or text messages. All students were also asked to refrain from exchanging information about the educational content presented in the research with friends and the students of other groups during the implementation of the project.

#### Scenarios

9 scenarios of professional belonging and 11 scenarios related to ethical behaviors were presented continuously to both intervention groups. The topics of the scenarios were written based on the experiences of the professors of the School of Nursing of Shiraz University of Medical Sciences, as well as professors specializing in professional ethics, and were approved by the research team.

Ethical scenarios and instruction for students included ethical topics, that is, moral dilemma and the importance of ethics in nursing; principles of ethics (principle of life value, principle of merit or liking, principle of justice or moderation, principle of honesty or truth-telling, principle of individual freedom); the four principles of bioethics (respect for independence and individual autonomy, non-maleficence, beneficence, justice; and important ethical issues in nursing (confidentiality, informed consent, nursing mistakes, ethical issues related to the beginning and end of life).

Topics of professional belonging scenarios included saving a life or obeying the law, receiving a gift in return for a job, responding to interdisciplinary discrimination, adhering to the law, confidentiality over a patient's life, reacting to co-worker misconduct, teacher behavior in internships, and professional nurses.

### Data collection procedure and instruments

Questionnaires were directly distributed by the researcher in all three stages of the test in all three groups. It took 20 to 30 min for each participant to complete the questionnaire. After completion, the questionnaires were coded based on intervention groups. Data collection tools in this study included demographic information form, a questionnaire to assess professional belonging of nursing students, and a questionnaire to assess nurses’ ethical behaviors.

#### Demographic information form

The form of demographic information includes age, gender, education, level of experience, and experience of participating in an e-learning method, which was prepared and adjusted according to the studies [45] and based on the opinions of experts in the research team.

#### Professional belonging assessment questionnaire

The professional belonging questionnaire in nursing students was designed by Zarshenas et al. (2018) with 47 items in 5 areas of individual perspective, professional acceptance, educational background, interprofessional communication, and messages perceived from the environment [46]. This questionnaire has two 5-part Likert scales of "strongly agree to strongly disagree" and "always to never". To score the questionnaire according to the semantic load of the items, a score of one is given to the scales "strongly disagree and never" and a score of 5 is assigned to the scales "strongly agree and always". For items whose semantic load is inverted, inverse scoring is performed. The questionnaire has 3 negative items. The minimum and maximum scores of the questionnaire are 47–235. Scoring levels are: very low level of belonging (85–47), poor level of belonging (122–86), average level of belonging (160–123), good level of belonging (197–161), and excellent level of belonging (235–198). The validity of this questionnaire was assessed, examining the content, face, and structural validity. Data validity was assessed using convergent validity (*r* = 0.6) and divergent validity (*r* = 0.1) and factor analysis. Finally, 47 items with a variance of 58.31% were explained in a 5-point Likert. Internal consistency with Cronbach's alpha was 0.97 and instrument stability was 0.76 [46].

#### Ethical behavior questionnaire

The questionnaire for assessing ethical behaviors based on codes of ethics in nurses has been designed by Momennasab et al. (2016) based on the ethics of nursing ethics in Iran [47]. This questionnaire has 26 items that are divided into 4 parts of "not at all" on a Likert scale. The scores range between 26–210, divided into 3 levels: weak ethical behaviors [22–26], moderate ethical behaviors (53–78), and good ethical behaviors (79–104). In general, the items include preserving their human dignity, adhering to professional obligations, being accountable, maintaining patients' privacy, promoting scientific and practical competence, and respecting individual independence. To determine the content validity, the questionnaire along with a copy of the Nursing Ethics Code was given to five professors of the School of Nursing and Midwifery, and necessary changes were made based on their comments. The validity of this questionnaire was assessed and confirmed using face validity (approval of experts) and content validity (0.95). In order to determine the reliability of the questionnaire, the test–retest method was used. 20 nursing students completed the questionnaires in two stages with a two-week interval, and its reliability was confirmed by correlation coefficient (*r* = 0.9) [47].

### Outcomes

The primary outcome is professional belonging, which was assessed by the professional belonging assessment questionnaire. Scores above 198 indicate an excellent level of belonging. Also, in ethical behaviors, which was evaluated by the Ethical Behavior Questionnaire, scores above 79 indicate good ethical behavior.

### Blinding

The present study was conducted in a single-blind way so that the participants did not know which group they were in. In order to prevent or reduce the communication of the participants with each other, the groups were selected from different semesters. Also, statistical analysis of data was performed by a statistician who was blind in terms of allocating data to learning groups.

### Ethical consideration

The present study was reviewed and approved by the ethics committee of Shiraz University of Medical Sciences with the number "IR.SUMS.REC.1396.156". It has also been registered in the Iranian Clinical Trial Registration Center (IRCT) with the registration number "IRCT20180612040063N1" and registration date "16/07/2018". All students participating in this study were given sufficient information about the objectives of this study. The students were explained that they can make comments and ask questions about how to conduct the study from the project implementers and that the data of this research will be kept completely confidential. They can also leave the study at any time if they wish so. After providing the candidates with this information, a form of informed consent was obtained from them. In addition, informed consent was obtained from the participants under the Helsinki Convention.

### Data analysis

The Statistical Package for the Social Sciences (SPSS) version 24 (SPSS Inc., Chicago, IL, USA) was employed for data analysis. To evaluate the demographic characteristics of the students, descriptive statistics of mean and standard deviation were used. Furthermore, a one-way ANOVA test was employed to compare the mean score of professional belonging and ethical behaviors of nursing students and to examine the differences between the 3 groups. Repeated measures test was employed to evaluate the effectiveness of the methods used on ethical behaviors. In all tests, the significance level was considered 0.05.

## Results

### Messages and feedback

In the e-portfolio group, students received the necessary feedback from the professors, which was a total of 188 comments and 51 feedback. A total of 435 messages and comments were recorded in the online conversation group.

### Sociodemographic characteristics

**Fig. 1:**
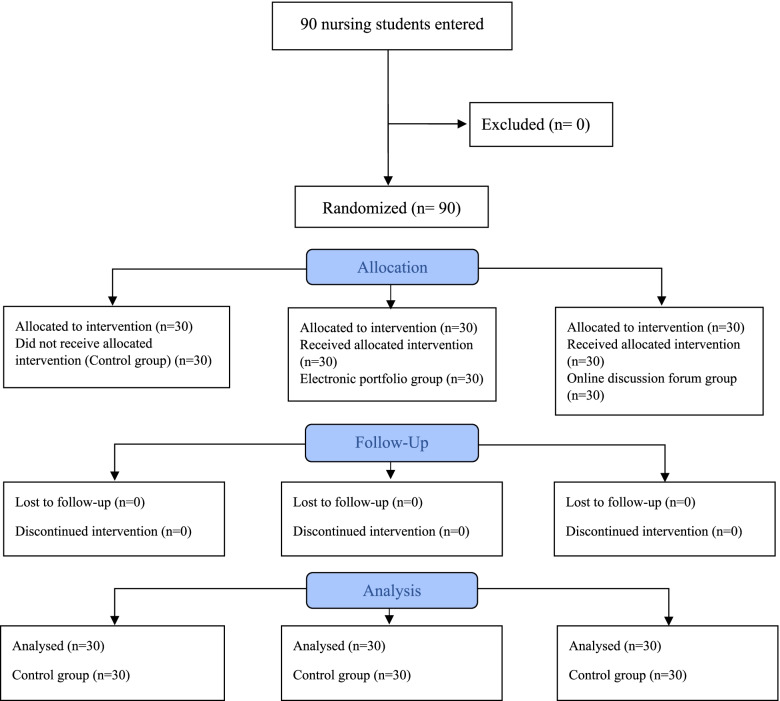
CONSORT flow diagram

According to the results, the mean age of participants in all groups was 33 ± 12, and 15 people (50%) from the control group, 16 people (53.3%) from the electronic portfolio group, and 16 people (53.3%) from the group of online conversation forum were women. More than 50% of the participants in all three groups were undergraduates. More than 50% of the students in the three groups had not studied professional belonging, while more than half of the students in all three groups had studied ethical behaviors. The results showed that 16 people in the control group (53.4%), 12 people (40%) in the electronic portfolio group and 9 people (30%) in the online conversation group had no work experience and more than half of the participants in each of the three groups had e-learning experience (Table 1, Fig. 1).

**Table 1:** Demographic characteristics of study participants by study groups

**Variables**		Control Group	Electronic Portfolio Group	Online Discussion Forum group
		**Number (%)**	**Number (%)**	**Number (%)**
**Gender**	Male	15(50)	14(46.7)	14(46.7)
	Female	15(50)	16(53.3)	16(53.3)
**Education**	Bachelor	21(70)	20(66.7)	20(66.7)
	Masters	9(30)	10(33.3)	10(33.3)
**Level of Experience**	No	16(53.4)	12(40)	9(30)
	Contract	7(23.3)	6(20)	9(30)
	Official personnel	7(23.3)	12(40)	12(40)
**Experience of Participating in an e-learning Method**	yes	10(33.3)	10(33.3)	13(43.3)
	No	20(66.7)	20(66.7)	17(66.7)

### Comparison of the mean score of professional belonging and ethical behaviors in the three stages of the test by study groups

According to the results, in the control group in all three stages of the experiment, there was no statistically significant difference in the mean scores of ethical behaviors (*P* value = 0.51) and professional belonging (*P* value = 0.57) (Table 2). However, there was a statistically significant difference in the mean scores of ethical behaviors (*P* value < 0.001) and professional belonging (*P* value < 0.001) in both e-portfolio and online conversation group groups in the three stages of the experiment. The mean scores of professional belongings in the e-portfolio group and the online discussion forum, immediately after the intervention were respectively 102.69 and 89.44, and four weeks after the intervention were 101.78 and 84.52, which had decreased compared to the pre-test in these two groups (103.40 and 109.20), (*P* value < 0.001). Given that the average score of professional belonging has decreased in both groups, the results of different dimensions of professional belonging need not be mentioned. Mean scores of ethical behaviors in the electronic portfolio group and online discussion forum, immediately after the intervention were respectively (99.9 and 101) and four weeks after the intervention were (101 and 104.2), which had increased compared to the pretest in these two groups (89.8 and 80.7) respectively, (*P* value < 0.001) (Table 2).

**Table 2:** Comparison of the mean score of professional belonging and ethical behavior in three stages of the test by study groups (*n* = 90)

**Study groups**	Variables	Mean ± SD			F	*P*-value
		**Pretest**	**Immediately after the intervention**	**Four weeks after the intervention**		
**Control group**	Professional belonging	107.66 ± 19.85	108.47 ± 18.90	108 ± 18.05	0.55	0.57
	Ethical behavior	90.8 ± 12.5	90.4 ± 11.2	90.8 ± 11.2	0.46	0.51
**Electronic Portfolio group**	Professional belonging	103.40 ± 19.17	102.69 ± 7.32	101.78 ± 5.91	13.88	< 0.001
	Ethical behavior	89.8 ± 11.8	99.9 ± 3.3	101 ± 2.2	17.9	< 0.001
**Online Discussion Forum group**	Professional belonging	109.20 ± 26.49	89.44 ± 9.66	84.52 ± 9.00	24.64	< 0.001
	Ethical behavior	80.7 ± 21	101 ± 4.4	104.2 ± 0.8	24.5	< 0.001

### Comparison of the mean score of professional belonging and ethical behaviors in the three study groups by test stage

As seen in Table 3, the results of the study showed that the mean of professional belonging (*P* = 0.57) and ethical behavior (*P* = 0.1) before the intervention did not differ between the intervention groups, so the three groups were not different in terms of mean score of professional belonging and ethical behavior at the beginning of the study.

**Table 3:** Comparison of the mean score of professional belonging in the three study groups by test stage (*n* = 90)

**Study groups**	Variables	Mean ± SD			F	*P*-value
		**Control group**	**Electronic Portfolio group**	**Online Discussion Forum group**		
**Pretest**	Professional belonging	108.20 ± 26.63	107.17 ± 26.69	111.28 ± 12.43	0.55	0.57
	Ethical behavior	90.8 ± 12.5	89.8 ± 11.8	80.7 ± 21	0.75	0.1
**Immediately after the intervention**	Professional belonging	108.18 ± 48.9	102.7 ± 7.32	89.9 ± 44.66	13.88	< 0.001
	Ethical behavior	90.4 ± 11.2	99.9 ± 3.3	101 ± 4.4	19.9	< 0.001
**Four weeks after the intervention**	Professional belonging	108.06 ± 18	101.5 ± 78.92	84.9 ± 5.01	24.60	< 0.001
	Ethical behavior	90.8 ± 11.2	101 ± 2.2	104.2 ± 0.8	27.1	< 0.001

In the stages immediately after the test and four weeks after the test, the comparison of the mean score of professional belonging in all three groups was significant (*P* < 0.001). The control group had a higher mean score of professional belonging immediately after the test (108.18 ± 48.9) and four weeks after the test (108.06 ± 18) compared to the other two groups (Table 3).

In the stages immediately after the test and 4 weeks after the test, the comparison of the mean score of ethical behavior in all three groups was significant (*P* < 0.001). The online discussion forum group had a higher mean score of ethical behavior immediately after the test (101 ± 4.4) and four weeks after the test (104.2 ± 0.8) compared to the other two groups (Table 3).

## Discussion

The COVID-19 pandemic has highlighted the need for e-learning. In the meantime, it has become much more important how to provide e-learning concepts such as professional belonging and ethical behaviors became much more important. The aim of this study was to compare the effectiveness of the two methods of the electronic portfolio and online discussion forum in teaching professional belonging and ethical behaviors to nursing students.

### Level of professional belonging of nursing students before, immediately after, and four weeks after the intervention in all 3 groups

According to the results, the mean of professional belonging before the intervention was not significantly different between the three study groups. Prior to the intervention in all three groups, professional belonging was at a low level. The study also revealed that in both groups of the intervention, professional belonging was reduced during the time. According to the professional belonging scores, there are no significant changes in the control group, while in the electronic portfolio and the online discussion forum groups, the scores have had a significant decreasing trend (poor level). In the online conversation forum group, students' professional belonging changed from a poor level to a very weak level after four weeks of intervention. These results can be explained by the abstract and challenging concept of professional belonging and the fact that the online discussion forum is an environment with many interactions, and therefore, learners have been influenced by each other more than by the content presented to them. Studies have also reported that too many interactions and high crowds of participants in the educational environment can negatively affect learning the content provided [48, 49].

Based on the results of a qualitative study by Zarshenas et al. (2017), the effective factors in the sense of professional belonging in nursing students include perceived messages such as community acceptance subsets, inner attitude, teacher feedback, work outcome, family acceptance, learning experiences, professional communication with the subsets of therapeutic communication, interpersonal communication of staff, interpersonal communication of professor with students in the department [50]. In a qualitative study, Ashktorab et al. (2017) found that the nature of belonging is divided into 5 concepts including moving in the direction of evolvement, attention to human and ethical values, professional integrity, achieving inner satisfaction, and the environment conformity with the learner [51]. Grobecker et al. (2016), investigating the relationship between professional belonging and perceived environmental stress in undergraduate internships in nursing students, contended that the concept of belonging as a basic human need positively affects the students’ self-confidence and learning motivation [52]. Sedgwick and Rougeau (2010), aiming to describe factors that affect nursing students' sense of belonging, showed that student belonging is influenced by interpersonal communication between students and other professional team members, patients, families, and the clinical environment. Evidence also suggested when nursing students feel that they are being treated as nurse colleagues, it has a particular effect on their sense of belonging [53]. A study by Bourgeois et al. (2011), describing a new model of clinical teaching and learning in nursing students, showed that students who feel part of the team and are encouraged by the staff feel supported. This sense of belonging affects students’ motivation and learning ability [54]. Other studies (Marsh et al. 2012; Brown et al. 2012; Sedgwick 2010) indicated that effective teacher-student communication and raising the level of students' motivation positively affects the formation of professional belonging [53, 55, 56].

As the effective factors in increasing the level of professional belonging are mentioned in the studies, the reason behind the decrease in the level of professional belonging in different stages of the present study may be the lack of adequate coverage of the factors affecting professional belonging by the online conversation forum and the electronic portfolio. Although in this environment there was an interaction between the professor and the student, this interaction has not been effective and sufficient in conveying the sense of teamwork and encouraging the student to belong to the profession.

### The level of ethical behaviors of nursing students before, immediately after, and four weeks after the intervention in all 3 groups

According to the results of the present study, the mean score of ethical behaviors in the three groups in the pre-test stage did not show a statistically significant difference. The results indicated that there was a statistically significant difference in the mean scores of ethical behaviors in the test in the e-portfolio group and the online discussion forum; which indicates an increase in the score level of ethical behaviors in two stages immediately after the intervention and four weeks after the intervention compared to the pre-test stage.

Searching different databases, the researchers did not find any study with the same topic in which the two methods of the electronic portfolio and online discussion forum were compared. Therefore, the studies that were somehow related to the main concepts of the present study were reviewed.

Many studies confirm the effect of education on learning the principles of nursing ethics. Chao et al. (2017), investigating the effects of a web-based education model in both experimental and control groups, implied that after completing the course, the experimental group had improved significantly in nursing ethical decision-making competencies, including skills in recognizing differences, comparing differences, actions, and identifying decision concepts compared to their performance before the course [57]. In a four-year study, Davis et al. (2009) concluded that the e-portfolio approach improves students' ability to promote ethical learning [58]. Garrett et al. (2013), evaluating the implementation of an electronic portfolio to evaluate clinical competencies in the undergraduate course of nursing, showed that the method is useful in improving the competencies of nursing students [59]. Choi et al. (2016) found that educational activities using a portfolio can increase personal focus and the ability of nursing students to better learn and update information [60]. These results were consistent with the findings of the present study.

In a study by Bahreini et al. (2012), investigating the development of nurses 'thinking skills after using the portfolio method, the results confirmed a positive effect of using the portfolio on the development of nurses' thinking skills [61]. Valizadeh et al.'s study suggested that a portfolio is a useful and effective way to increase learning and cognitive skills [62]. Some studies showed that teaching ethical principles can be effective in promoting moral sensitivity (Imanifar et al. 2015; Izadi et al. 2013; Hassanpoor et al. 2011) [63–65]. Although none of these studies examined the effect of using the electronic portfolio method on students' ethical behaviors, from the perspective that in these studies the portfolio has been used as an intervention method for nurses or nursing students, and they also point out the effectiveness of the portfolio in different ways, their findings can be in line with some of the results of the present study.

We found that in the portfolio group and also in the online discussion forum group, there is a statistically significant relationship between the mean score in the three stages of the test and the mean score immediately after the intervention and four weeks after the intervention in both groups (electronic portfolio and online discussion forum) while in the control group, without any intervention, no increase in score was observed in three stages of the experiment. Raghavan et al. (2010) believed that online conversation communities are one of the most popular tools for supporting student communication and collaboration in web-based learning environments, and one of the best ways to share ideas is to receive feedback, send problems and comment on other students' posts [66]. Hudson et al. (2014), who have considered teaching the concepts of nursing ethics through the online discussion forum, found that online discussion forum has beneficial results in learning and promoting the concepts of nursing ethics, and the method can be adopted as an educational strategy with positive potential for nursing concepts courses. The online education strategies employed in the conversation forum can enhance dialogue, reflection, collaboration, and building knowledge of the nursing students to enter the clinic [67]. These results are in line with the findings of our study.

Also, it can be said that the results of the present study are in line with Park et al. (2012) [68] and Vahedian azimi et al. (2008) [69]. These researchers found that training courses for nursing students lead to the promotion of nursing students' ethical reasoning, which is in line with the results of the present study. On the other hand, Goethals et al. (1992) claimed that nurses, despite being taught ethics in training courses, had a lower level of ethical reasoning after entering the clinical environment [70]. This is not consistent with the results of the present study. The difference can be due to different teaching methods and research environments. In the present study, the participants included undergraduate students of the 5th semester and above and postgraduate students of the 2nd semester and above, in other words, the students had entered the clinical environment, and an improvement in ethical behavior was observed in this study.

### Strength, limitations, and future research directions

This is the first study that investigates and compares two e-learning methods as well as teaching the two concepts of professional belonging and ethical behaviors in virtual environments. Also, the present study simultaneously examines two approaches as well as individual and collective training in the form of electronic portfolios and online discussion forums. We employed only one software (Mahara) to separate the groups, and there was no need to use multiple virtual software.

One of the limitations of this study is the limited time of the intervention. Thus, longer follow-up of the subjects is suggested in future studies. Also, considering the concept of creative behaviors and professional belonging and its areas and considering that the present study was conducted only in one academic center, for future studies, we suggest investigating the problem in several universities in different cultures. On the other hand, due to the limitations of the present study in considering the factors in creating a sense of professional belonging in the virtual education system, further research are needed to study all factors which are effective in creating a sense of professional belonging for training in a virtual or face-to-face learning environment.

## Conclusion

The findings of the study suggest that education through two methods of the electronic portfolio and online discussion forum can be effective and useful in improving the level of ethical behaviors in students. In other words, our research emphasizes the need to implement educational methods based on two methods of electronic portfolio and online discussion forum to improve and promote ethical behaviors. Therefore, considering the valuable concept of ethical behaviors in nursing and due to the undesirable observance of ethical principles, it is necessary to intervene to improve and promote these concepts to maintain the professional identity of nursing. On the other hand, the study showed that the use of virtual methods (electronic portfolio and online discussion forum) leads to a decrease in the level of professional belonging. Therefore, it can be acknowledged that these two virtual education systems have been inefficient in implementing and transmitting the concepts of some of the effective factors in creating a sense of professional belonging in students. This decrease can be attributed to the widespread and unfamiliar concept of professional belonging to students. Therefore, further work needs to be conducted with a greater focus on areas of professional belonging. It is also suggested that effective strategies be adopted to increase the level of professional belonging in a clinical setting with the presence of professors.

## Data Availability

The datasets used during the current study are available from the corresponding author on reasonable request.

## Abbreviations

E-portfolio: Electronic Portfolio
E-learning: Electronic Learning
